# Fecal microbiota transplantation combined with ruxolitinib as a salvage treatment for intestinal steroid-refractory acute GVHD

**DOI:** 10.1186/s40164-022-00350-6

**Published:** 2022-11-09

**Authors:** Yin Liu, Ye Zhao, Jiaqian Qi, Xiao Ma, Xiaofei Qi, Depei Wu, Yang Xu

**Affiliations:** 1 National Clinical Research Center for Hematologic Diseases, Jiangsu Institute of Hematology, Key Laboratory of Thrombosis and Hemostasis of Ministry of Health, The First Affiliated Hospital of Soochow University, 188 Shizi Street, Suzhou, Jiangsu Province China; 2 Institute of Blood and Marrow Transplantation, Collaborative Innovation Center of Hematology, Soochow University, Suzhou, China; 3 Soochow Hopes Hematonosis Hospital, Suzhou, Jiangsu Province China

**Keywords:** HSCT, FMT, Ruxolitinib, Steroid-refractory GVHD, Intestinal GVHD

## Abstract

Acute graft-versus-host disease (aGVHD), especially intestinal aGVHD, is one of the most severe complications after allogeneic hematopoietic stem cell transplantation (HSCT). Fecal microbiota transplantation (FMT) has been applied to the treatment of intestinal steroid-refractory aGVHD (SR-aGVHD). Ruxolitinib is the first drug recommended for SR-aGVHD. Here, we reported the outcome data from 21 patients who had received the combined treatment of FMT with ruxolitinib as a salvage treatment in intestinal SR-aGVHD after HSCT. The overall response rate on day 28 was 71.4% (95% CI 50.4–92.5%), including 10 patients with complete responses. The durable overall response at day 56 in responders was 80%. GVHD relapse rate was 33.3% in responders. The levels of inflammatory cytokines as well as T cells and NK cells activation declined. The diversity of the intestinal microbiota was improved in responders. Viral reactivations and severe cytopenia were the major adverse events (61.9% and 81% respectively). The estimated 6-month overall survival was 57.1% (95% CI: 35.9–78.3%), while event-free survival was 52.4% (95% CI: 21.7%-64.1%). Collectively, FMT with ruxolitinib could be an effective treatment for intestinal SR-aGVHD after HSCT.

*Trial registration*: ClinicalTrials.gov identifier: NCT03148743.

**Supplementary Information:**

The online version contains supplementary material available at 10.1186/s40164-022-00350-6.

To the Editor,

Acute graft-versus-host disease (aGVHD), especially intestinal localization, remains one of the most unremovable barriers to the success of allogeneic hematopoietic stem cell transplantation (HSCT), leading to late morbidity and mortality. Fecal microbiota transplantation (FMT) was reported to be effective [1], but attempts have been made to explore combination treatments with other drugs to increase response rate and improve survival of intestinal steroid-refractory aGVHD (SR-aGVHD). The initial experience reported by Bilinski et al. [2], as well as the clinical trial (*ClinicalTrials.gov ID: NCT04269850*) sponsored by St. Petersburg State Pavlov Medical University [3], gave some evidence to show the potential efficiency of the combined treatment with FMT and ruxolitinib. We previously conducted a phase 1 clinical trial of FMT as a therapeutic option for intestinal GVHD (*ClinicalTrials.gov ID: NCT03148743*) [4–6] and mentioned a subset of patients using ruxolitinib with a higher response rate. Herein, we reported this subset showing the efficacy of combined treatment of FMT with ruxolitinib as a salvage treatment in intestinal SR-aGVHD after HSCT.

A cohort of 21 patients was treated with FMT and the add-on ruxolitinib as an additional therapy for grade III–IV intestinal SR-aGVHD after HSCT between November 2017 and July 2019. The inclusion criteria included: (1) age from 12 to 60; (2) stable state of disease; (3) grade III–IV intestinal aGVHD; 4) steroid-refractory GVHD defined as the following: disease progression based on organ assessment after at least 3 days of high-dose methylprednisolone (MP) at 2 mg/kg/d, a lack of response without change in visceral GVHD (absence of partial response or better) after 5 days, or exacerbation of acute GVHD or treatment failure during MP taper. Follow-up was continued through July 2019 before the analysis started. The exclusion criteria were uncontrolled infections; inability to swallow tablets and severe organ damage due to reasons other than GVHD. Once diagnosed with steroid-refractory GVHD, patients were given the combined treatment of FMT 2 capsules three times daily for 2–3 days, including 40–50 mg microbiota per full therapy, and oral ruxolitinib with an initial dose of 5 mg twice daily. The median delay between initiation of ruxolitinib and first FMT was 4 days, while the max was 11 days. Frozen fecal microbiota was obtained from China fmtBank (Nanjing, China) and transferred into capsules for swallowing. Our study obtained ethics approval from the Ethics Review Committee of our institution and was conducted in accordance with the Declaration of Helsinki. All patients were provided with written informed consent for participation.

GVHD grading was assessed routinely using standardized criteria per MAGIC guidelines [7]. Peripheral blood counts, infections, and virus reactivations were also monitored closely. Treatment responses were defined as complete response (CR), partial response (PR), or treatment failure (NR). A CR was defined as the absence of any symptoms related to GVHD. A PR was defined as the improvement of at least one grade in the severity of aGVHD in at least one site and without deterioration in any other organ. Treatment failure was defined as the absence of improvement, deterioration in any organ, or the development of new GVHD symptoms. Overall response rate (ORR), durable overall response (DOR), time to first response, overall survival (OS), event-free survival (EFS), malignancy relapse rate, GVHD relapse rate, and treatment-related adverse events were assessed. Levels of cytokines and the percentages of lymphocytes were measured before treatment and at the best response time.

A cohort of 21 patients, average of 29 years old (range: 15–59), received FMT plus ruxolitinib as a further treatment for grade III-IV intestinal SR-aGVHD in our center between November 2017 and July 2019 (Table 1). Most enrolled patients manifested GVHD in at least 2 organs, including skin rash (11/21, 52.4%) and elevated bilirubin (10/21, 47.6%), despite enterit

**Table 1:** Patient and treatment characteristics

#	Age (years)	Gender	Diagnose	Disease status	Donor source	Conditioning regimen	Stem cell source	GVHD Prophylaxis	Onset of aGVHD after HSCT
1	44	Male	AML	CR	MUD(O → AB)	Bu/Cy	PB	CsA + MMF + MTX + ATG	22
2	59	Male	AML	Relapse	HRD(A → A)	CBA	BM + PB	CsA + MMF + MTX + ATG	19
3	34	Male	AML	CR	HRD(O → B)	Bu/Cy	BM + PB	CsA + MMF + MTX + ATG	19
4	29	Female	AML	Refractory	HRD(B → B)	Bu/Cy	BM + PB	CsA + MMF + MTX + ATG	10
5	40	Male	AML	Relapse	HRD(B → A)	Bu/Cy	BM + PB	CsA + MMF + MTX + ATG	30
6	18	Male	SAA	NR	HRD(A → B)	Bu/Cy	BM + PB	CsA + MMF + MTX + ATG	20
7	29	Male	AML	CR	HRD(B → B)	Bu/Cy	BM + PB	CsA + MMF + MTX + ATG	30
8	18	Female	ALL	CR	HRD(B → B)	Bu/Cy	BM + PB	CsA + MMF + MTX + ATG	25
9	47	Male	AML	CR	HRD(O → A)	Bu/Cy	BM + PB	CsA + MMF + MTX + ATG	97
10	21	Female	ALL	CR	HRD(O → B)	Bu/Cy	BM + PB	CsA + MMF + MTX + ATG	42
11	28	Female	AML	CR	HRD(A → A)	Bu/Cy	BM + PB	FK506 + MMF + MTX + ATG	22
12	47	Male	MDS	PD	HRD(B → O)	Bu/Cy	BM + PB	CsA + MMF + MTX + ATG	17
13	16	Male	SAA	NR	HRD(AB → AB)	Bu/Cy	BM + PB	CsA + MMF + MTX + ATG	18
14	23	Female	AML	CR	HRD(A → A)	Bu/Cy	BM + PB	CsA + MMF + MTX + ATG	22
15	38	Male	AML	CR	HRD(B → B)	Bu/Cy	PB	CsA + MMF + MTX + ATG	55
16	15	Female	MDS	NR	HRD(B → B)	Bu/Cy	BM + PB	CsA + MMF + MTX + ATG	24
17	25	Male	AML	Relapse	HRD(O → B)	Bu/Cy	BM + PB	CsA + MMF + MTX + ATG	51
18	45	Male	HAL	CR	HRD(O → O)	Bu/Cy	BM + PB	CsA + MMF + MTX + ATG	100
19	16	Male	ALL	Refractory	HRD(A → A)	Bu/Cy	BM + PB	CsA + MMF + MTX + ATG	28
20	26	Male	MDS	PD	HRD(AB → AB)	Bu/Cy	BM + PB	CsA + MMF + MTX + ATG	23
21	54	Male	AML	CR	HRD(O → O)	Bu/Cy	BM + PB	CsA + MMF + MTX + ATG	22

#	Additional organ involvement to intestine manifestations	aGVHD grade	Response to combined treatment	Virus reactivation after combined treatment	Infection after combined treatment	Severe cytopenia	Last fellow-up (days after response)	Status	GVHD relapse (severity, days after response)
1	None	III	NR	CMV	–	3	111	Dead	–
2	Skin and liver	IV	NR	–	–	4	40	Dead	–
3	Skin	IV	PR	–	–	4	127	Dead	Intestine (1, + 47d)
4	Skin and liver	IV	NR	–	–	4	22	Dead	–
5	Skin and liver	III	PR	CMV	–	–	73	Dead	–
6	None	III	CR	CMV enteritis	–	–	677	Alive	–
7	Skin and liver	III	PR	–	–	4	661	Alive	Skin, intestine (2, + 88d)
8	Skin	III	PR	CMV enteritis, EBV	–	–	685	Alive	–
9	None	III	CR	Urinary polyomavirus	Intestinal infection	3	707	Alive	Intestine (2, + 226d)
10	Skin and liver	IV	NR	–	Intestinal infection Fungal pneumonia	4	173	Dead	–
11	Liver	IV	NR	CMV	Sepsis	4	53	Dead	–
12	None	III	NR	CMV	Pneumonia	4	28	Dead	–
13	Liver	III	CR	CMV	–	4	713	Alive	Liver (1,+42d)
14	None	III	CR	CMV	–	–	31	Dead	–
15	None	III	CR	CMV retinitis	–	4	821	Alive	–
16	Skin	III	CR	–	–	3	527	Alive	–
17	Skin and liver	IV	CR	–	–	4	693	Alive	Skin, liver (2,+144d)
18	None	III	CR	CMV	–	3	485	Alive	–
19	Skin and liver	IV	PR	–	Pneumonia	4	524	Alive	–
20	Skin and liver	IV	CR	CMV + EBV	–	4	458	Alive	–
21	None	III	CR	CMV	Intestinal infection	4	384	Alive	–

*HSCT* hematopoietic stem cell transplantation, *aGHVD* acute graft-versus-host disease, *AML* acute myelogenous leukemia, *MDS* myelodysplastic syndrome, *ALL* acute lymphoblastic leukemia, *SAA* severe aplastic anemia, *HAL* hybrid acute leukemia, *CR* complete response, *NR* no response, *PD* progressive disease, *MUD* matched unrelated donor, *HRD* haplo-identical related donor, *BM* bone marrow, *PB* peripheral blood, *CSA* cyclosporine A, *MMF* mycophenolate mofetil, *MTX* methotrexate, *FK506* tacrolimus, *ATG* anti-thymocyte globulin

is with diarrhea. Their characteristics and therapy-related profiles are shown in Table 1 and Fig. 1A.

**Fig. 1:**
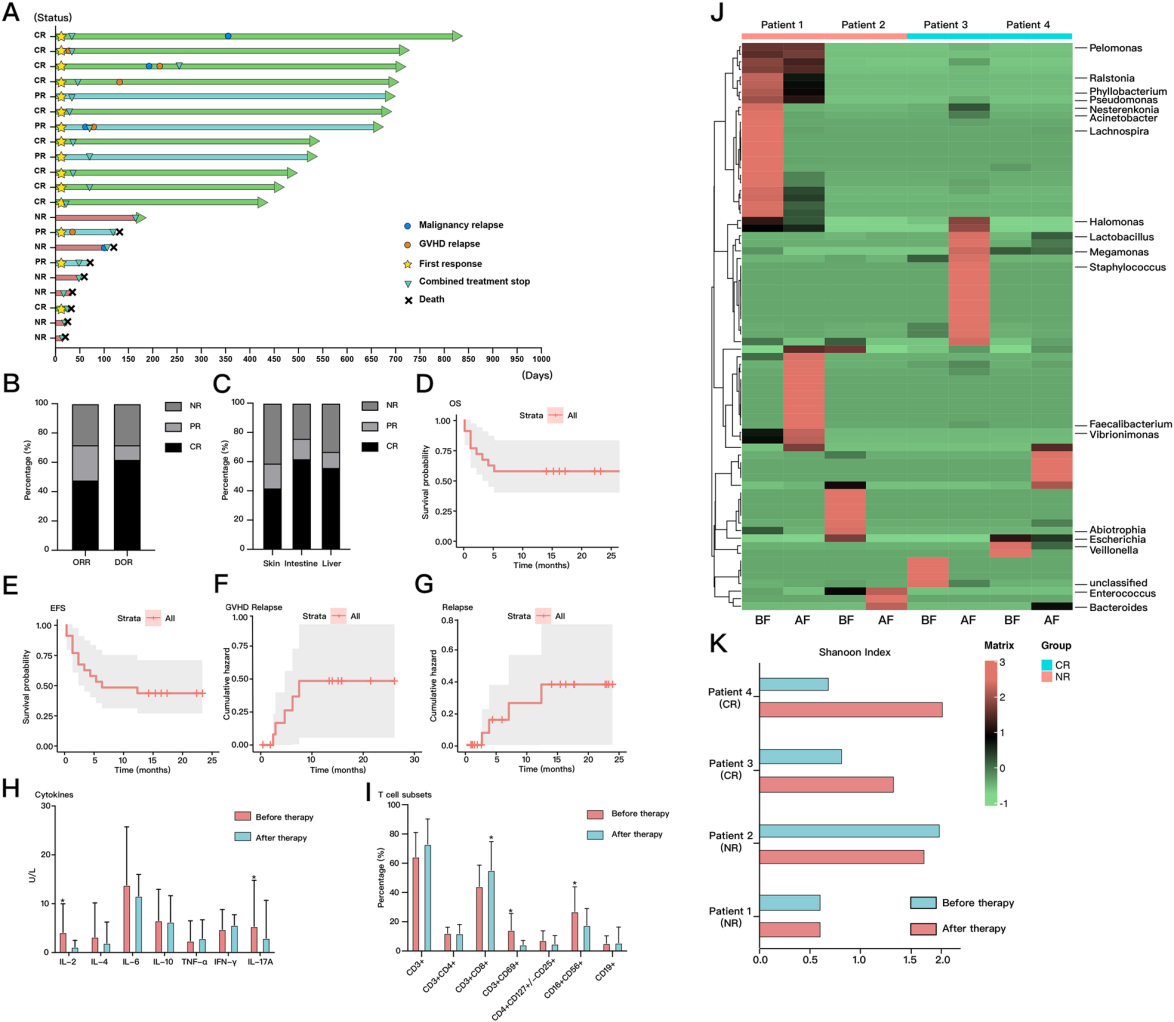
Acute GVHD therapy profile and treatment response. **A** Long-term outcomes of 21 patients with intestinal SR-aGVHD after the initiation of fecal microbiota transplantation combined with ruxolitinib. **B** The primary endpoint was the overall response (complete response or partial response) on day 28, and the key secondary endpoint was the durable overall response on day 56. **C** The best response to combination treatment in intestinal SR-aGVHD patients was demonstrated in different target organs. **D** The overall survival of all patients treated with combination treatment for SR-aGVHD. **E** The event-free survival of all patients treated with combination treatment for SR-aGVHD. **F** The cumulative incidence of aGVHD relapse in responsive patients; the competing risks were transplant-related mortality and relapse. **G** The cumulative incidence of malignancy relapse in all patients; the competing risk was transplant-related mortality. **H** Comparison of IL-2, IL-4, IL-6, IL-10, TNF-α, IFN-γ, and IL-17A levels detected in peripheral blood between baseline and within 3 weeks of the initiation of ruxolitinib treatment. The levels of IL-2 and IL-17A significantly declined. **I** Relative percentages of T cell subsets were examined before and during the use of ruxolitinib in peripheral blood by flow cytometry. The percentage of activated T cells (CD3^+^CD69^+^ cells) cells and NK cells (CD16^+^CD56^+^ cells) significantly decreased after the combination treatment. **J** The composition of the gut microbiota in four selected patients before and after the combination of ruxolitinib and FMT at the genus level. Patients 1 and 2 failed to respond to the treatment, while patients 3 and 4 showed complete responses. **K** The Shannon index of gut microbiota was measured to demonstrate the diversity of the intestinal microbiota before and after the combination of ruxolitinib and FMT. A restoration of diversity with a higher Shannon index was observed in CRs

The ORR on day 28 was 71.4% (95% CI 50.4–92.5%), including 10 CRs and 5 PRs, with a median time of 10 days to achieve the first response. The DOR at day 56 in responders was 80% (Fig. 1B). The median duration of follow-up was 15.7 months. The median duration of steroid tapering to half dose was 14 days. A higher overall response rate (76.2%) was observed in patients with intestinal involvement among distinct target organs (Fig. 1C). The estimated 6-month OS was 57.1% (95% CI: 35.9–78.3%), while EFS was 52.4% (95% CI: 21.7%-64.1%) (Fig. 1D, E). GVHD relapse rate was 33.3% in responders, among whom three patients experienced chronic GVHD (Fig. 1F). Meanwhile, malignancy relapse was observed in four patients at the last follow-up (Fig. 1G). Viral reactivations (61.9%), bacterial infections (28.6%), and severe cytopenia (grades 3–4, 81%) were the most frequent adverse events observed in our study.

In inflammatory cytokines analysis, we observed significant declines in IL-2 and IL-17A and similar trends in IL-4, IL-6, and IL-10 following the combined treatment compared to the baseline values (Fig. 1H). Additionally, the percentage of activated T cells and NK cells decreased at the same time (Fig. 1I). We collected data on the temporal microbiota dynamics of four patients (two CRs and NRs respectively). The percentage of beneficial bacteria, such as *Lactobacillus*, increased in CRs; whereas *Escherichia*, which was reported to be strongly correlated with GVHD in a mouse model, reduced after the treatment (Fig. 1J) [1, 8, 9]. The diversity of the intestinal microbiota was improved in responders, with an apparent increase in the Shannon index (Fig. 1K). Furthermore, the levels of inflammatory cytokines and the percentages of activated T cells declined, while regulatory T cells increased in answer to the combined treatment when compared to the baseline levels in patient 3 with a CR (Additional file 1: Figure S1 C, D).

Our study further underlined the additive effect of FMT with ruxolitinib in the salvage treatment of intestinal SR-aGVHD, supported by a high ORR of 71.4% and impressive outcomes. Randomized controlled trials are needed to be conducted to demonstrate the efficiency and safety of the combined treatment. We hope the modifications of protocols for combined treatment with FMT and ruxolitinib could be taken into consideration.

## Supplementary Information

**Additional file 1: Figure S1.** Comparison of inflammatory cytokines and T cell subsets in the four selected patients. **A** Comparison of IL-2, IL-4, IL-6, IL-10, TNF-α, IFN-γ, and IL-17A levels detected in peripheral blood between baseline and the following 4 weeks after the initiation of combined treatment in Patient 1 with no response. The level of cytokines showed no significant change at 3 weeks and extraordinarily increased at the last follow-up, ending with death. **B** Relative percentages of T cell subsets were examined before and 4 weeks after the initiation of combination therapy in peripheral blood by flow cytometry in Patient 1 with no response. The percentage of CD3+CD69+ cells showed no difference, while the percentage of CD4+CD127+/-CD25+ cells declined to zero. **C** Comparison of IL-2, IL-4, IL-6, IL-10, TNF-α, IFN-γ, and IL-17A levels detected in peripheral blood between baseline and the following 4 weeks after the initiation of combined treatment in Patient 3 with a complete response. The level of cytokines significantly declined. **D** Relative percentages of T cell subsets were examined before and 4 weeks after the initiation of combination therapy in peripheral blood by flow cytometry in Patient 3 with a complete response. The percentage of CD3+CD69+ cells declined coincidently, while the percentage of CD4+CD127+/-CD25+ cells increased to a remarkably high level.

## Data Availability

The datasets used and analyzed during the current study are available from the corresponding author upon reasonable request.

## Abbreviations

aGVHD: Acute graft-versus-host disease
HSCT: Hematopoietic stem cell transplantation
SR-aGVHD: Steroid-refractory aGVHD
FMT: Fecal microbiota transplantation
MP: Methylprednisolone
ORR: Overall response rate
DOR: Durable overall response
OS: Overall survival
EFS: Event-free survival
CR: Complete response
PR: Partial response
